# A model for non-monotonic intensity coding

**DOI:** 10.1098/rsos.150120

**Published:** 2015-05-06

**Authors:** Johannes Nehrkorn, Hiromu Tanimoto, Andreas V. M. Herz, Ayse Yarali

**Affiliations:** 1 Department of Biology II, Bernstein Center for Computational Neuroscience Munich and Graduate School of Systemic Neurosciences, Ludwig-Maximilians-Universität München, Martinsried 82152, Germany; 2 Max Planck Institute of Neurobiology, Martinsried 82152, Germany; 3 Tohoku University Graduate School of Life Sciences, Sendai 980-8577, Japan; 4 Research Group Molecular Systems Biology of Learning, Leibniz Institute for Neurobiology, Magdeburg 39118, Germany; 5 Center for Brain and Behavioural Sciences, Magdeburg, Germany

**Keywords:** associative learning, homeostatic plasticity, neural coding, olfaction, stimulus intensity

## Abstract

Peripheral neurons of most sensory systems increase their response with increasing stimulus intensity. Behavioural responses, however, can be specific to some intermediate intensity level whose particular value might be innate or associatively learned. Learning such a preference requires an adjustable trans- formation from a monotonic stimulus representation at the sensory periphery to a non-monotonic representation for the motor command. How do neural systems accomplish this task? We tackle this general question focusing on odour-intensity learning in the fruit fly, whose first- and second-order olfactory neurons show monotonic stimulus–response curves. Nevertheless, flies form associative memories specific to particular trained odour intensities. Thus, downstream of the first two olfactory processing layers, odour intensity must be re-coded to enable intensity-specific associative learning. We present a minimal, feed-forward, three-layer circuit, which implements the required transformation by combining excitation, inhibition, and, as a decisive third element, homeostatic plasticity. Key features of this circuit motif are consistent with the known architecture and physiology of the fly olfactory system, whereas alternative mechanisms are either not composed of simple, scalable building blocks or not compatible with physiological observations. The simplicity of the circuit and the robustness of its function under parameter changes make this computational motif an attractive candidate for tuneable non-monotonic intensity coding.

## 2. Introduction

Varying a sensory stimulus can influence behaviour in two fundamentally different ways. First, the map from stimulus to behaviour can be one-to-one. For example, the reaction time of human beings to a light stimulus decreases steadily with increasing light intensity [1]. At the neuronal level, monotonic stimulus–response curves suffice to explain this phenomenon. Second, a particular behaviour may only be triggered by a certain range of intermediate stimulus values; for instance, rats and fruit flies prefer weak, but not strong, salt solutions over plain water [2,3]. In this case, the brain needs to represent the stimulus in a non-monotonic way to generate the appropriate behaviour.

For some stimulus attributes, bell-shaped tuning curves at the sensory periphery solve this task. The peaked frequency tuning of hair cells [4], for example arises because the basal membrane of the vertebrate cochlea vibrates most strongly at a location determined by the frequency of the presented sound. For other stimulus dimensions, such as sound amplitude [5], sensory neurons have monotonic input–output curves, raising the question of how non-monotonic stimulus dependencies of behavioural responses are generated.

A suitable system to study this general question is odour-intensity learning. Odour intensity is typically encoded in a monotonic way by the first- and second-order olfactory neurons; consequently, the neuronal population activated by an odour grows with increasing odour intensity and the representation of a lower intensity is nested within that of a higher intensity (e.g. [6–8]). The overall increase in neuronal activation with rising odour intensity can be useful to explain the ability to detect odour gradients (as argued, e.g. in [9–11]) as well as the improvement of olfactory detection, associative learning and memory retrieval at higher intensities (e.g. [12]). Changes in the hedonic value of an odour with increasing intensity can arise if neurons with different sensitivities are connected to opponent downstream pathways (e.g. [13]). Finally, changes in discriminability across odours with rising intensity are consistent with growing odour representations (e.g. [14]). However, a key behavioural observation remains unexplained: animals form associative memories specific to trained odour intensities such that later on, neither lower nor higher intensities release as strong a conditioned behaviour, as shown in the fruit fly [12,15–17], honeybee [18] and mouse [19]. This intensity specificity of learning suggests that along the olfactory pathway, downstream of the initial monotonic encoding, odour intensity must be re-coded in a non-monotonic manner.

We present a simple, biologically plausible neuronal circuit motif that does just this. We quantitatively compare the intensity coding ability of this model to the intensity specificity of olfactory memories, as assayed in the fruit fly and discuss how this circuit may be implemented in the fly olfactory system, thus leading to experimentally testable hypotheses. The circuit motif found may also be relevant for other cases where stimulus intensity must be encoded in a non-monotonic fashion to enable intensity-specific behaviours (for an example in the auditory modality, see [5]).

## 3. Material and methods

### 3.1 Input layer

The activity of excitatory and inhibitory input neurons (figure 2*b*) are described by logistic input–output functions:

3.1 $${\text{exc}}_{k}(i)=\frac{1}{e^{-4b(i-a_{k})}+1}\;\text{and}\;\text{inh}(i)=\frac{{\mathrm{inh}}_{\max}}{e^{-4b_{\mathrm{inh}}(i-a_{\mathrm{inh}})}+1},$$

where *i* is the odour intensity in logarithmic units. Thus, *a*_*k*_ and *a*_inh_ are the odour intensities at the turning points of the respective logistic sensitivity functions, i.e. a large negative *a*-value implies a high sensitivity. The factor 4 in the exponents is chosen so that *b* and *b*_inh_ are the slopes at the turning points, where *b*>*b*_inh_. The parameter inh_max_>1 scales the sensitivity function of the inhibitory input neuron. For simplicity, only three excitatory input neurons are considered. Their *a*_*k*_ values are shifted in steps of one logarithmic unit.

### 3.2 Intermediate layer

The activity of the intermediate-layer neurons (figure 2*c*) are calculated as rectified weighted sums of the input activities as

3.2 $${\text{inter}}_{k}(i)=\text{Rect}(w_{\mathrm{exc}}\;{\text{exc}}_{k}(i)+w_{\mathrm{inh}}\;\text{inh}(i)),$$

where *w*_exc_ and *w*_inh_ are the weights of the respective excitatory and inhibitory inputs. The rectifying function Rect(*x*) is defined as Rect(*x*<0)=0 and Rect(*x*≥0)=*x*, and results in a threshold neuronal activation function.

### 3.3 Homeostatic plasticity

We consider two scenarios for homeostatic plasticity. In both cases, we do not model how the synaptic strength changes in response to each individual stimulus presentation but rather calculate the resulting mean effect of homeostatic plasticity under the assumption that already prior to the specific associative odour-shock training, the system has been exposed to odours drawn from a broad range of concentrations.

In the first scenario (figure 3*a*(i)), the weights of the inhibitory synapses to the intermediate layer are set uniformly to be *w*_inh_=−1; whereas each excitatory synapse (*w*_exc_) is subject to homeostatic plasticity. To implement the mean effect of this regulatory process, the weights *w*_exc_ are adjusted based on the sensitivity of the respective excitatory input neurons: the more sensitive an input neuron is (more negative *a*-value), the higher its mean activation and, consequently, the mean rate at which it drives the downstream intermediate-layer neuron. This effect will be balanced by homeostatic plasticity. As a measure of the input neuron's sensitivity, we take the integral of the input–output function exc_*k*_(*i*) over a concentration range [*c*_0_, *c*_1_]:

3.3 $$s=\int_{c_{0}}^{c_{1}}\text{exc}(i)\;\text{d}i=\frac{1}{4b}\ln\left\{\frac{1+e^{4b(c_{1}-a)}}{1+e^{4b(c_{0}-a)}}\right\}=s(a).$$

The sensitivity function *s*(*a*) approaches *c*_1_–*c*_0_ for a→−∞ and zero for a→∞. For intermediate values *c*_0_<*a*<*c*_1_, *s*(*a*) scales roughly linear in *a*. Then, based on *s*(*a*), we adjust the respective excitatory output weight as

3.4 $$w_{\mathrm{exc}}(a)=-\alpha (s(a)-d),$$

where *α* is a scaling factor and *d* is set such that *w*_exc_(*a*)>0 (see inset in figure 3*a*(i)). Thus, in the spirit of homeostatic plasticity, the more sensitive an excitatory input neuron is, the weaker its synapse to the intermediate layer will be. Mechanistically, this could either be implemented through ‘local’ homeostatic plasticity [20,21] acting directly at this excitatory synapse, or through classical homeostatic plasticity [22], as we only consider a single excitatory input to each intermediate-layer neuron.

In the second scenario (figure 3*b*(i)), the weights of all excitatory synapses are set to *w*_exc_=1. Implementing the mean effect of homeostatic plasticity, the weight of each inhibitory synapse is scaled according to the sensitivity of the cognate excitatory input neuron as

3.5 $$w_{\mathrm{inh}}(a)=-\tilde{\alpha }s(a),$$

where $\tilde{\alpha }$ is a scaling factor (see inset in figure 3*b*(i)). Thus, the smaller the excitatory drive of an intermediate neuron, the weaker is also its inhibitory input, in accordance with experimental findings on homeostatic plasticity at inhibitory synapses [21,23,24].

### 3.4 Output neuron and associative plasticity

The activity of the output neuron is calculated as the weighted sum of the intermediate-layer neuron activities:

3.6 $$\text{out}(i)=\sum_{k}w_{\mathrm{training},k}{\text{inter}}_{k}(i).$$

Initially, the weights *w*_training,*k*_ are all zero. During associative odour-shock training (e.g. figures 2*d*, 3*a*(iii),*b*(iii)), these weights change proportional to the odour-induced activity in the respective intermediate neuron, owing to the delivery of a concurrent reinforcement signal as

3.7 $$\Delta w_{\mathrm{training},k}=\Theta (\text{shock})\;{\text{inter}}_{k}(i_{\mathrm{training}}),$$

where *θ*(*x*) is the Heaviside function, defined as 0 if *x*≤0 and 1 otherwise, representing the presence versus absence of shock and *i*_training_ is the odour intensity at training.

## 4. Results

### 4.1 Olfactory memories of flies are odour-intensity specific

**Figure 1.**
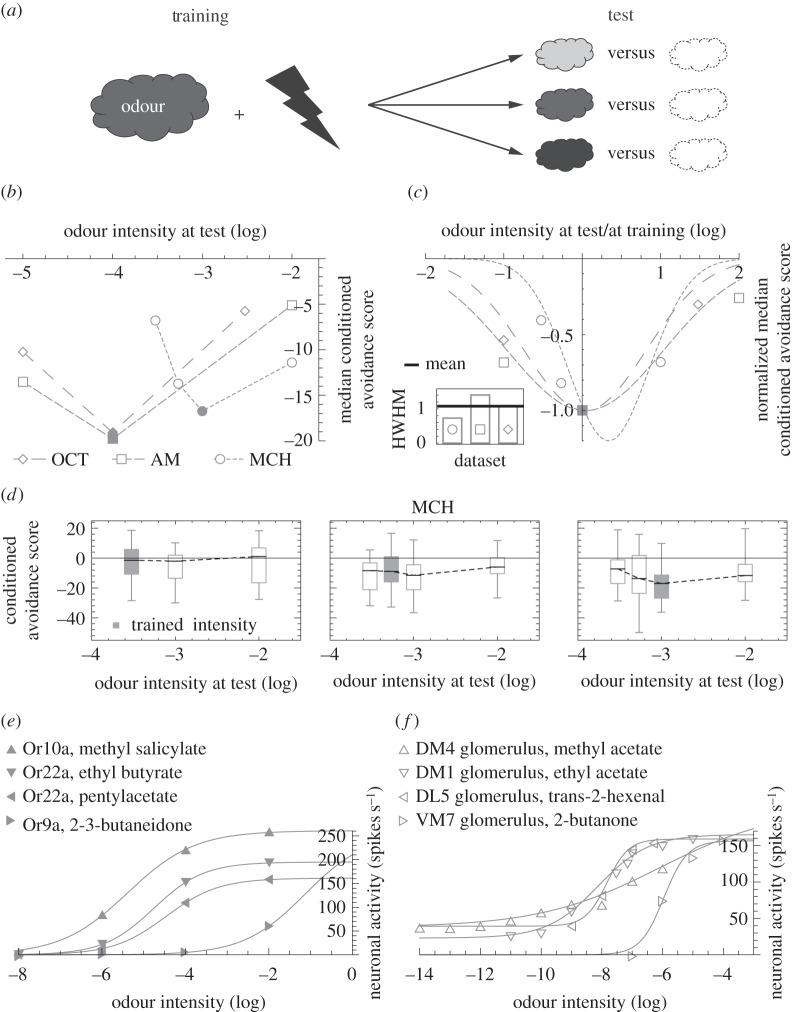
(*Overleaf*.) Learned olfactory behaviour is intensity specific, unlike the response characteristics of sensory and projection neurons. (*a*) One subgroup of flies is trained *en masse* such that an odour is temporally paired with electric shock; whereas a second subgroup (not sketched) is presented with odour and shock in an unpaired fashion. Each subgroup is then tested for choice between the trained odour versus a non-odorous solvent and a preference index is calculated as PI=(#_Odour_−#_Solvent_)⋅100/#_Total_, where # is the number of flies on each side. A conditioned avoidance score is defined as CAS=(PI_Paired_−PI_Unpaired_)/2, i.e. PI_Unpaired_ acts as a baseline to which PI_Paired_ is compared. Negative CASs indicate conditioned avoidance. To probe for the intensity specificity of the conditioned behaviour, we compare CASs across groups, which are trained with one common odour intensity, but tested with different intensities (different grey shades). (*b*) In three different experiments, the design in (*a*) is applied to the odours 3-octanol (OCT), *n*-amylacetate (AM) and 4-methlycyclohexanol (MCH). Critically, odour intensities are chosen from the dynamic range of the respective dose–response curves of learning and retrieval [12]. The median CAS is shown as a function of the odour intensity at test. For filled symbols, the testing intensity equals training intensity. Sample sizes are left to right *N*=20, 20, 20 for OCT, *N*=20, 24, 24 for AM and *N*=24, 31, 24, 24 for MCH, referring to the number of independent measurements. Data are from [12]. For a more detailed description of the methods, see [12]. (*c*) Data in (*b*) are normalized along the intensity axis by dividing test intensities by the training value; and along the CAS axis by dividing median CASs by values from matching training and testing intensities. The results are fitted with Gaussian distributions. Their half widths at half maximum (HWHM, inset) are similar (mean: 1.1, s.d.: 0.3) and close to results from odour-sugar associative learning experiments in larval *Drosophila* ([17], HWHM mean±s.d.=1.5±0.4). (*d*) In three different experiments, the design in (*a*) is applied to the odour MCH. In each experiment, a different MCH intensity is used for training. The box plots represent the median by the midline, 25 and 75% by the box boundaries and 0 and 100% by the whiskers. Grey filling indicates matching training and test intensities. Training with a very low MCH intensity (left panel) results in CASs that are not different from zero, no matter the testing intensity (Kruskal–Wallis test: *H*=1.04, d.f.=2, *p*=0.59; one-sample sign test comparing pooled data to zero: *p*=0.90; *N*=16, 24, 24). When the training intensity is somewhat raised (middle panel), the CASs statistically do not depend on test intensity and when pooled indicate slight conditioned avoidance (Kruskal–Wallis test: *H*=4.65, d.f.=3, *p*=0.20; one-sample sign test comparing pooled data to zero: *p*<0.05; *N*=31, 33, 33, 33). Finally, for a further raised training intensity (right panel), CASs depend on test intensity (Kruskal–Wallis test: *H*=9.27, d.f.=3, *p*=0.02) and are strongest when training and test intensities resemble each other (Mann–Whitney *U* tests: test at 0.0003 versus 0.001: *U*=147.00, *p*<0.05/3; test at 0.00054 versus 0.001, *U*=290.00, *p*=0.17; test at 0.01 versus 0.001, *U*=159.00, *p*<0.05/3; *N*=24, 31, 24, 24). Data in the right panel from [12]. Note that the training intensity used in this panel is chosen from the middle of the dynamic range of the dose–effect function for learning and retrieval [12]. (*e*) Monotonic intensity tuning of single olfactory sensory neurons (OSN) which ectopically express the specified olfactory receptor (Or) molecule, taken from [27]. For a comparison between the electrophysiology of such transgenic OSNs versus wild-type ones, see [28]. Note that monotonic intensity tuning has been documented also with respect to wild-type OSNs (e.g. [29]). (*f*) Monotonic intensity tuning of single olfactory projection neurons, innervating the indicated antennal lobe glomeruli, taken from [29]. In (*e*) and (*f*), the lines correspond to fitted logistic functions.

The intensity specificity of fruit fly olfactory memories has been reported in several studies using different developmental stages, experimental rationale and reinforcers ([12,15–17,25,26]; for a comparative discussion see [12]). We start with a meta-analysis of three experiments that apply a common paradigm to three odours [12]. In each experiment, flies are trained *en masse*, with pairings of a chosen intensity of the respective odour and electric shock. Different groups of flies are then tested for their avoidance of this odour at the trained, a lower or a higher intensity (figure 1*a*). In each case, conditioned avoidance is scored by comparing the behaviour of flies trained as explained with paired presentation of odour and shock versus flies trained with temporally unpaired presentation of the same stimuli; thus, the scores refer to effects of associative learning and not to innate odour-responsiveness. Across all three experiments, flies show the strongest conditioned avoidance when the testing and training intensities match (figure 1*b*). For better comparison across experiments, we align the three datasets along the stimulus and the response axes and find similar Gaussian fits, despite the diversity of odours (figure 1*c*). Results from an appetitive olfactory learning assay in *Drosophila* larvae [17] paint a similar picture (see the legend of figure 1*c*).

### 4.2 A simple circuit motif for odour-intensity-specific memories

Fruit fly olfactory sensory neurons (OSNs) and projection neurons (PNs) increase their activity with rising odour intensity at the single-cell level, as exemplified in figure 1*e*,*f* (see also [8,10,27,29–32] for demonstration of this property using a variety of methods). As a direct consequence of such monotonic input–output curves, an odour at low intensity excites relatively few neurons, whereas the same odour at a higher intensity recruits not only these but also additional neurons. Based on such a nested representation of odour intensities alone, the memory trace of a low-intensity odour would be activated at least as strongly by a higher intensity of the same odour. However, olfactory associative memories in flies are intensity specific (figure 1*a*–*c*). This implies that non-monotonic intensity responses must emerge in downstream layers of the olfactory pathway. The following model accomplishes this task.

**Figure 2.**
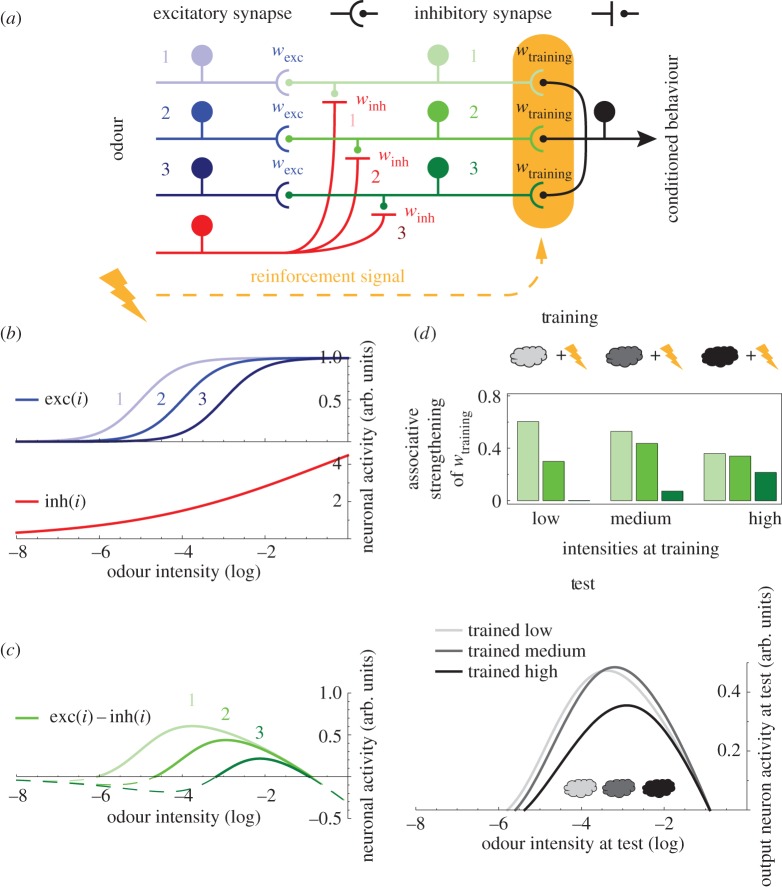
Convergence of excitatory and inhibitory inputs generates non-monotonic intensity tuning but does not allow intensity-specific memories. (*a*) Model circuit. The odour is feed-forwardly processed through three neuronal layers. For simplicity, the input layer consists of only three excitatory (blue) and one inhibitory neuron (red). The excitatory neurons connect one-to-one with three intermediate-layer neurons (green) with weights *w*_exc_, the single inhibitory neuron provides input to all intermediate neurons with weights *w*_inh_. Intermediate neurons converge onto one output neuron (black) with weights *w*_training_. The output synapses of the intermediate layer also receive an electric shock-induced reinforcement signal (yellow). (*b*) The activity of input neurons increases with increasing odour intensity according to the logistic functions exc(*i*) (blue) and inh(*i*) (red), respectively. The different exc(*i*) share slope and asymptote, but are shifted along the intensity axis. Critically, the function inh(*i*) is less steep than the exc(*i*) functions. (*c*) The activity of each intermediate neuron is the weighted difference between its cognate excitatory input and the shared inhibitory input, i.e. exc(*i*)–inh(*i*) (green), as the weights of all inputs are adjusted to 1. The resulting bell-shaped tuning curves are nested. (*d*) Using this circuit, we simulate three experiments. Different odour intensities (differently shaded clouds) are paired with shock during training. Upon presentation of the respective odour intensity, each intermediate neuron is activated depending on its tuning. Upon the contingent delivery of shock, a reinforcement signal strengthens intermediate-layer output synapses, proportional to the pre-synaptic activity level (green bars). At test, we present the circuit with a series of odour intensities, including the trained ones, and measure the activity of the output neuron, which indeed depends non-monotonically on odour intensity in each experiment (grey lines). Critically, however, the activity peaks around the same intensity-range in all three experiments, despite the difference in the training intensities used.

The input layer of the model harbours multiple excitatory neurons (figure 2*a*, blue) with different, monotonic responses, represented by logistic functions that are shifted by different offsets along the stimulus axis (figure 2*b*, blue). These functions are inspired by fly OSN- and PN-electrophysiology (e.g. [27] and [29]; figure 1*e*,*f*), as well as computational models of olfactory transduction (e.g. [33]). The modelled excitatory input neurons are connected one-to-one with neurons of the intermediate layer (figure 2*a*, green). In addition, a single inhibitory neuron with monotonic input–output function (figure 2*a*,*b*, red) provides input to all intermediate neurons. The convergence of excitation and inhibition endows each intermediate neuron with a bell-shaped tuning curve (figure 2*c*). The relative shift of sensitivity across the excitatory input neurons (figure 2*b*, blue) and the shallower sensitivity curve of the inhibitory neuron as compared with the excitatory neurons cause the intermediate neurons to differ in their tuning curves (figure 2*c*) but the nestedness of these tuning curves rules out that memories are intensity specific.

To illustrate this important limitation we introduce an output neuron, onto which all intermediate neurons converge (figure 2*a*, black). Prior to any training, the synaptic weights are set to zero so that the output neuron does not respond to even the most intense odour (note that the innate olfactory behaviour pathway is not represented in the model). When we train the circuit by pairing a given odour intensity with electric shock (figure 2*d*, training), each intermediate neuron is activated to a certain degree, which depends on its tuning curve and the intensity of the presented odour. In addition, a reinforcement signal, induced by the electric shock is delivered to the output synapse of each intermediate neuron (figure 2*a*, yellow). Owing to this reinforcement signal, each output synapse is strengthened proportional to the respective level of odour-induced activity (figure 2*d*, training). This potentiation of output synapses is the trace for the odour-shock memory. To read out this trace at test, we present the circuit with various odour intensities and measure the activity of the output neuron (figure 2*d*, test). If the circuit mimics the flies' intensity-specific learning (figure 1*a*–*c*), the output neuron will respond most strongly when the training and testing intensities match. This is not the case (figure 2*d*, test): although after all three kinds of training, the output neuron activity depends on the odour intensity with a bell-shaped function, the peaks do not correspond to the respective odour intensities used at training.

**Figure 3.**
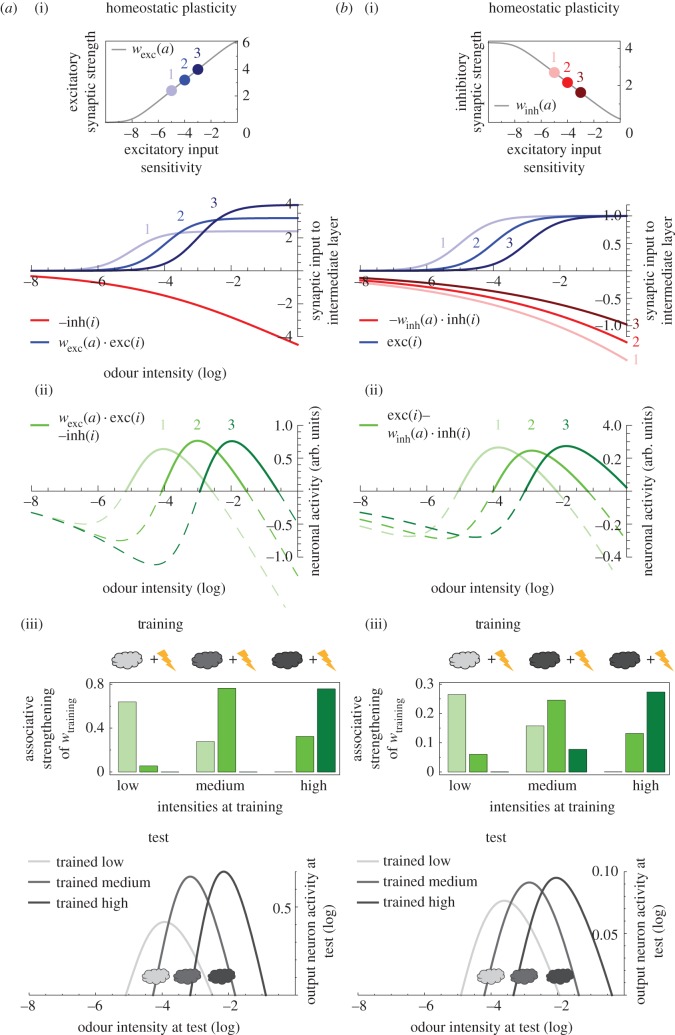
Adding homeostatic plasticity enables intensity-specific memories. (*a*) Homeostatic plasticity is added to the circuit motif of figure 2: each excitatory synapse onto the intermediate layer is adjusted based on the respective input neuron's sensitivity. Thus, the weight *w*_exc_ becomes a function of *a*, which is the turning point of the logistic function exc(*i*), such that a large *a*-value (indicating a low neuronal sensitivity) is counterbalanced by a higher *w*_exc_ and vice versa (*a*(i) inset). (*a*(i)) Consequence of this homeostatic regulation on the excitatory synaptic inputs to the intermediate layer (*w*_exc_(*a*)⋅exc(*i*), blue), together with the unadjusted inhibitory input (−inh(*i*), red). (*a*(ii)) The convergence of these inputs confer non-monotonic and non-nested intensity tuning to the intermediate neurons (*w*_exc_(*a*)⋅exc(*i*)–inh(*i*), green). In (*a*(iii)), we apply the training and test design from figure 2*d*. Unlike in figure 2*d*, the output neuron at test responds most strongly to the respective intensity used at training. (*b*) With an alternative homeostatic plasticity rule, each inhibitory input synapse is adjusted based on the sensitivity of the cognate excitatory input. Thus, *w*_inh_ becomes a function of *a* as shown in the inset of (*b*(i)). In (*b*(i)), we plot the resulting, homeostatically regulated inhibitory inputs to the intermediate layer (−*w*_inh_(*a*)⋅ inh(*i*); red) and the excitatory inputs (exc(*i*), blue). (*b*(ii)) These converging inputs confer non-monotonic and non-nested tuning curves to the intermediate-layer neurons (exc(*i*)–*w*_inh_(*a*)⋅inh(*i*), green). In (*b*(iii)), we apply the training-test design from figure 2*d* and obtain results similar to those in (*a*(iii)).

To solve this problem, we enrich the circuit shown in figure 2*a* with homeostatic synaptic plasticity, which maintains the activity level of intermediate-layer neurons within a certain range by boosting weak signals, while suppressing strong ones [20–24]. Indeed, homeostatic regulation has been shown on different levels of the fly olfactory system [34–36]. Accordingly, we assume that the animal is exposed to a wide range of odours at various concentrations prior to the associative odour-shock training, and that this leads to homeostatic plasticity, which we describe at the level of time-averaged effects in our model. First, we implement this mechanism at the excitatory input synapses projecting onto the intermediate layer (figure 3*a*(i)). For simplicity, we calculate the resulting mean effect of homeostatic regulation instead of modelling the synaptic strength changes in response to each individual stimulus presentation. As expected, the more often an intermediate-layer neuron is activated on average (because of a more sensitive input neuron), the weaker the respective excitatory synapse becomes. In an alternative scenario (figure 3*b*(i)), we implement the homeostatic plasticity at the inhibitory synapses onto the intermediate layer. This means that the inhibitory synapses are adjusted such that the more often an intermediate-layer neuron is activated, the stronger the respective local inhibitory synapse becomes. Both scenarios result in non-monotonic and, critically, non-nested tuning curves across the intermediate layer (figure 3*a*(ii), *b*(ii)). Consequently, in either scenario, when we train the circuit with a given odour intensity paired with electric shock, the output neuron indeed responds most strongly to this very intensity at test (figure 3*a*(iii),*b*(iii)), mimicking the flies' odour-intensity-specific memories. Furthermore, with lower training intensities, the output neuron activity at test is smaller and more broadly tuned (especially pronounced in figure 3*a*(iii)), reflecting fly behavioural data, where lower training intensities result in weaker and less intensity-specific conditioned avoidance scores (figure 1*d*).

**Figure 4.**
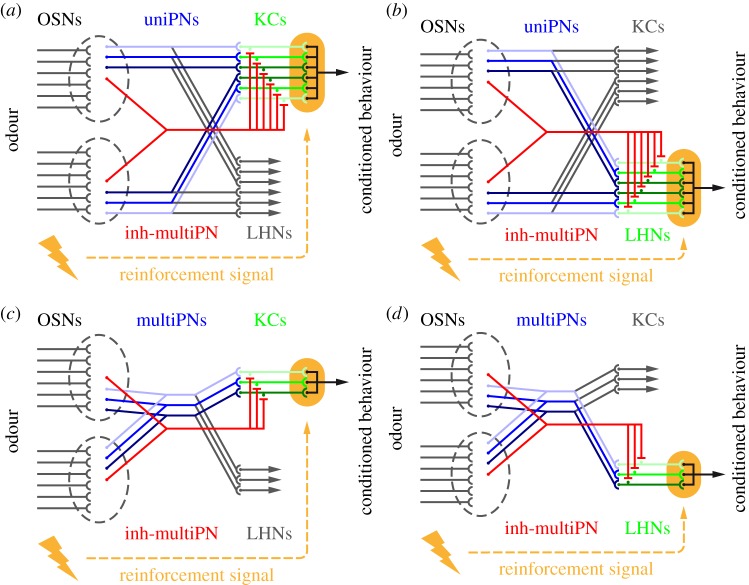
Scenarios for implementation in the fly olfactory system. We present four scenarios for implementing the circuit motif in figure 2*a* in the adult *Drosophila* olfactory system. In adult flies, approximately 1300 olfactory sensory neurons (OSNs) per hemisphere converge onto approximately 50 antennal lobe glomeruli, based on their olfactory receptor expression [37,38]. For simplicity, in each scenario, we depict only two glomeruli (grey dashed ellipsoids), each with six afferent OSNs. On average three homotypic uniPNs receive input at each glomerulus [39]. In (*a*) and (*b*), we use these ‘sister’ uniPNs as the excitatory input neurons (blue) and consider as the intermediate layer (highlighted in green) their post-synaptic partners, either the mushroom body Kenyon cells (KCs) in (*a*) or the lateral horn neurons (LHNs) in (*b*). For implementing the feed-forward inhibition from antennal lobe to the KCs (*a*) or to the LHNs (*b*), we propose anatomically described GABAergic multi-glomerular projection neurons (inh-multiPNs; red; see the main text for details). As the responses of uniPNs are partially odour identity-specific, an entanglement of identity and intensity coding is conceived in (*a*) and (*b*). Alternatively, the coding of identity and intensity may be segregated. Thus, in two further scenarios, we use multiPNs as the excitatory input neurons (multiPNs; blue; see the main text for anatomical references) and take as the intermediate layer their downstream partners the KCs (*c*) or the LHNs (*d*). The inhibitory channels are as proposed for (*a*) and (*b*), respectively. Note that these scenarios are based on adult *Drosophila* olfactory anatomy. At the larval stage, each antennal lobe glomerulus is innervated by a single uniPN [40] thus (*a*) or (*b*) would not apply; whereas (*c*) or (*d*) may be plausible as multi-glomerular projection neurons have been described in the larval olfactory system [41].

Within a biological implementation of the model, the sketched excitatory neurons of the input layer need to receive common olfactory input so that the rank order of sensitivities does not change with odour identity. This circuit property can be fulfilled if these neurons were, e.g. uni-glomerular projection neurons innervating a common antennal lobe glomerulus, or multi-glomerular projection neurons each innervating a large sum of glomeruli (see Discussion and figure 4 for details). Note also that the time scale on which the homeostatic plasticity occurs is long compared to the time scale of the training and testing of odour-intensity-specific memories. This is consistent with homeostatic adjustments taking place during development and/or early life in response to olfactory exposure.

Importantly, the ability of the model to mimic flies' intensity coding is robust across a large parameter space, as revealed by a detailed sensitivity analysis (electronic supplementary material, figure S1). This flexibility may render the model circuit attractive for a variety of neuronal systems.

## 5. Discussion

The early stages of most sensory systems encode stimulus intensity with monotonic response curves (see [42,43] for vision, [44] for hearing, [45] for somatosensation, [46] for taste, but [3] for an exception regarding taste). The absence of non-monotonic intensity tuning in the initial processing steps is particularly striking in olfaction, because animals readily form odour-intensity-specific associative memories [12,15–19,25,26].

A suitable case to tackle this discrepancy is the fruit fly, where olfactory sensory and projection neurons (OSNs and PNs) have monotonic responses [8,10,27,29–32] (but see [9] for few examples of non-monotonic tuning at PN output sites). Peaked intensity tuning curves mainly emerge downstream, in mushroom body (MB) Kenyon cells (KCs) [47,48], and, as recently found, in lateral horn neurons (LHNs) [49] (also see [50] for locust KCs). What kind of connectivity carries out this transformation?

### 5.1 Computational models of non-monotonic intensity coding

In a model suggested by Luo *et al.* [51], a layer of uni-glomerular projection neurons (uniPNs) gives randomly connected excitatory output to a layer of 2500 KCs and drives a global inhibitory neuron that also impinges upon the KCs. The synapses along the feed-forward inhibitory pathway are adjusted such that more sensitive uniPNs contribute stronger to inhibition, whereas those KCs with stronger excitatory input receive more inhibition. Although the model is concerned with the odour identity-tuned responses of third-order olfactory neurons, a substantial portion of the KCs also shows peaked intensity tuning. The large number of KCs in adult flies (e.g. [52]) fulfils the model's requirement. In flies, however, KCs display a heterogeneity of function in supporting short- versus long-term and perhaps even appetitive- versus aversive olfactory memories (e.g. [53,54]) and they support a variety of additional behavioural functions (e.g. context-generalization [55], regulation of sleep [56], decision-making [57]). It is thus not clear how many KCs would be ‘available’ for implementing this solution with respect to a given olfactory learning event. The randomness of the connectivity in Luo *et al.*'s model [51], despite being realistic (e.g. [58]), does not allow pinpointing an explicit minimal network structure that transforms monotonic responses to non-monotonic ones. Such information would be useful for generalizing to other developmental stages, species and modalities, where fewer neurons might be available. Technical implementations, too, would benefit from a design principle, which can be scaled according to need.

Here, different from Luo *et al*. [51] or the related framework of reservoir computing [59], we explicitly suggest a dedicated circuit motif transforming monotonic into non-monotonic responses. In this three-layer feed-forward circuit (figure 2), the input layer consists of multiple excitatory neurons with monotonic response curves reflecting different sensitivities and a single inhibitory neuron with a monotonic response function that is less steep than those of the excitatory neurons. Convergence of these elements onto the intermediate layer results in peaked tuning curves. To support intensity-specific memories, however, it is critical to have different neurons with non-nested tuning curves peaking at different values along the intensity axis (figures 2 versus 3). This property is generated through a homeostatic adjustment [20–24] of the excitatory versus inhibitory balance in the input to the intermediate neuron layer. Finally, all intermediate-layer neurons converge onto a single output neuron, with synapses that are subject to associative plasticity. In this scheme, the generation of peaked tuning curves in the intermediate layer resembles the mechanism by which mammalian auditory brainstem neurons encode sound amplitude with bell-shaped profiles [5]. The necessity of homeostatic plasticity, on the other hand, echoes a key ingredient of Luo *et al*.'s [51] model, where the feed-forward inhibitory pathway synapses are adjusted to balance out the effects of the excitatory pathway.

Could there be even simpler circuit motifs than those considered so far? Indeed, endowing intermediate-layer neurons with a resonate-and-fire mechanism [60] could achieve the required nonlinear transformation between stimulus intensity and circuit output. As in our model framework, odour intensity would be encoded monotonically in the frequency of periodic spike trains of input neurons (e.g. PNs). If their discharge frequency is too low or too high, the intermediate neuron would not fire. However, if the discharge frequency is close to (or matches) the resonance frequency of the intermediate neuron, this neuron would generate periodic spike trains, too.

Now consider a larger group of resonate-and-fire neurons with different resonance frequencies whose range covers the behaviourally relevant firing rates of the input neurons. In an odour-shock training episode with a given odour intensity, there would thus only be one neuron or a small group of neurons in resonance with the input. Different odour intensities would drive different subgroups. Owing to a reinforcement signal, the output synapses of these activated intermediate neurons would be associatively strengthened, laying down an intensity-specific memory trace. The larger the mismatch between input frequency and resonance frequency, the smaller the synaptic changes would be. After learning, only odour intensities close to the trained intensity would be transmitted; whereas lower or higher intensities would be filtered out, thus enabling intensity-specific conditioned behaviour.

A decisive ingredient of this alternative mechanism is the layer of resonate-and-fire neurons with cell-specific resonance frequencies. From each of these neurons, one would expect that during odour presentation, the inter-spike-intervals cluster around a particular value, reflecting the neuron's inverse resonance frequency. Neither fly [47,61], nor locust [50,62] KCs show this property; instead, KC inter-spike intervals vary significantly within a single response to one odour, across multiple responses to the same odour and across responses to different odours. The data presented in [49] suggest that the same is true for LHNs.

A further alternative model [17] is conceptually similar to our approach but assigns to each intermediate neuron one excitatory and one inhibitory pre-synaptic partner, thus employing more neurons than the present model and requiring a rather specific circuit structure that may not match the fly olfactory system. The model proposed here is simpler and could be readily implemented in the fly olfactory system, where activity-dependent homeostatic plasticity has been observed at various neuropils [34–36].

### 5.2 Implementation of the present circuit motif in the *Drosophila* olfactory system

Given the scarceness of odour-intensity-resolved physiological data from the MB [47,48,50] and the LH [49] and the absence of behavioural studies that discriminatively test for the roles of these in intensity learning, we believe it too early to restrict the discussion to one or the other neuropil. We thus consider all known neuron types in the fly olfactory system to suggest four alternative implementations of the model, including detailed references to anatomy, thus pointing to testable hypotheses (figure 4).

In the first two scenarios (figure 4*a*,*b*), the excitatory input neurons for each antennal lobe glomerulus correspond to the homotypic uniPNs innervating that glomerulus [39]. UniPNs have monotonic response functions (see above for references). To accommodate our model, we assume different sensitivities for the different uniPNs innervating a common glomerulus; as these receive common OSN-input, the rank order of their sensitivities will be identical for all odours. As uniPNs project to both the MB and the LH, we consider either neuropil as the intermediate layer in figure 4*a*,*b*, respectively. Feed-forward inhibition from antennal lobe to MB is limited. The single identified type of likely GABAergic, multi-glomerular PN (inh-multiPN) projecting to the MB calyx ([39]; named ‘mlPN4’ in [63]) could implement the inhibition in figure 4*a*. Alternatively, inhibition could be carried out by the APL neuron, which provides feedback to the KCs [63,64] or by the MB-C1 neuron which connects LH to the MB [52] and has GABAergic counterparts in other insects [65,66]. For the inhibitory channel in figure 4*b*, several GABAergic inh-multiPN types projecting to the LH (e.g. ‘mlPN2’ and ‘-3’ in [63]) are available as candidates. Interestingly, at least one of these neurons innervates almost the entire antennal lobe (‘mlPN3’ in [63]; also see [67]), providing a particularly elegant solution. Furthermore, some inh-multiPN types are known to have monotonic sensitivity functions, as required for a role in the present model [68–70]. Whether the detailed parametric properties of these sensitivity functions would enable non-monotonic intensity coding across the behaviourally relevant range remains to be investigated. As the responses of uniPNs are partially odour identity-specific, the scenarios in figure 4*a*,*b* conceive an entanglement of identity and intensity coding. This is especially true for the scenario in figure 4*a*, as a large body of evidence point to the MB KC output synapses as the site of the critical plasticity underlying learned olfactory behaviour (reviewed in [71,72]; also see e.g. [54,73,74] for neurogenetic analyses in *Drosophila* and e.g. [75,76] for electrophysiological accounts in other insects).

The extent to which animals should discriminate versus generalize along the odour intensity and the odour identity dimensions probably depends on the behavioural task at hand. In that sense, separating, instead of entangling the coding of these dimensions offers more degrees of freedom in regulating the balance between discrimination and generalization. Thus, in two further scenarios (figure 4*c*,*d*), we use multiPNs as the excitatory input neurons. Two anatomical candidates for such neurons project, respectively, to the MB and the LH and to only the LH (‘mPN4’ and ‘lPN2’ in [63]). Accordingly, we consider either neuropil for the intermediate layer in figure 4*c*,*d*, respectively, with the inhibitory channels as elaborated above. In these two scenarios, as the intensity coding circuit sums up activity across the antennal lobe glomeruli, it loses the information on odour identity and these two dimensions are coded separately. Whether and how these stimulus dimensions can be bound together to form a unitary percept of a particular odour at a particular intensity to enable an intensity- and identity-specific olfactory memory remains open.

The scenarios we propose can be directly tested because they are based on identified neuron types, as detailed above, and because transgenic tools for interfering with these neurons are available (e.g. [63]). The experimental design outlined in figure 1*a* can be used in conjunction with these tools to investigate the roles of specific neuron types. Particularly, the role of multi-glomerular projection neurons have so far been considered in the framework of innate olfactory behaviour, given their monotonic intensity sensitivity (e.g. [69,70]); a role for these neurons in enabling non-monotonic coding and associative learning of intensity is a novel suggestion.

Behavioural responses often depend in a bell-shaped fashion on certain stimulus attributes whose preferred value might be determined through associative learning. The present model demonstrates that an elementary circuit motif can achieve the required tuneable signal transformation. The model can be tested experimentally at a quantitative level. Owing to its simplicity, the underlying circuit motif could serve as an attractive candidate for tuneable non-monotonic intensity coding.

## Supplementary Material

Figure S1

## Supplementary Material

Figure S1. Supplementary figure legend

## Data accessibility

The data plotted in figure 1*b*,*d* at Dryad: http://datadryad.org/ (doi:10.5061/dryad.qk0b0)
