# Supplementary figures and images for: A model for non-monotonic intensity coding
Source: R Soc Open Sci. 2015 May 6;2(5):150120. doi: 10.1098/rsos.150120 (PMC4453257; doi:10.1098/rsos.150120)

A

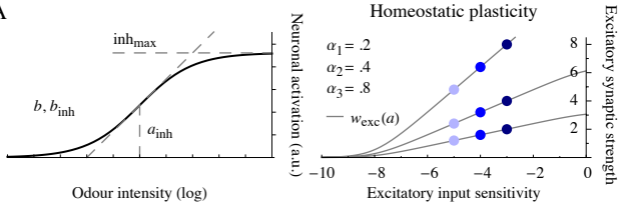

B

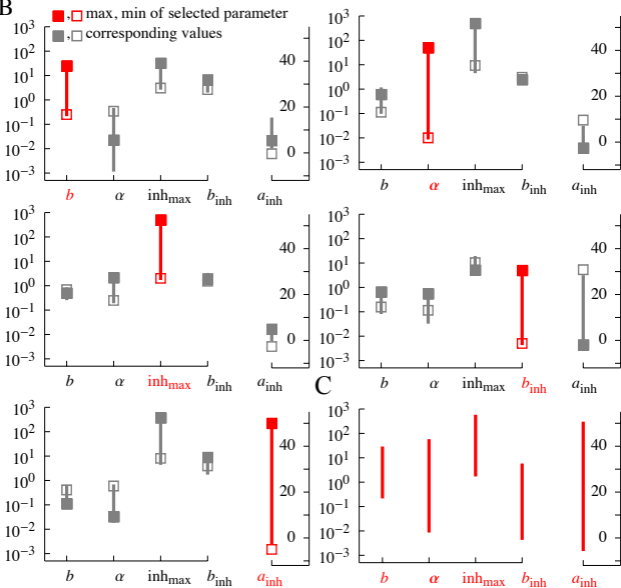

Supplement: Figure S1 [file rsos150120supp1.pdf]
