# Supplementary material for: A model for non-monotonic intensity coding
Source: R Soc Open Sci. 2015 May 6;2(5):150120. doi: 10.1098/rsos.150120 (PMC4453257; doi:10.1098/rsos.150120)
Supplement: Figure S1. Supplementary figure legend [file rsos150120supp2.docx]

**Supplementary figure legend**

**Figure S1. The model supports intensity-specific memories across a wide range of parameters**

(A) For a sensitivity analysis, based on the scenario in Figure 3A, we consider the parameters *a*_inh_ and *b*_inh_, denoting the intensity value and slope at the turning point of inh(*i*); inh_max_, the asymptotic activity of the inhibitory neuron; and *b*, the slope at the turning points of exc(*i*). In addition, parameter *α* characterizes the potency of homeostatic plasticity, i.e., as *α* is raised, the *w*_exc_(*a*) becomes steeper, emphasizing the difference in strengths between the excitatory synapses onto the intermediate layer (blue). (B) We repeat the experiment in Figure 3Aiii using only medium odour intensity (10^-3^) for training. We take the half width at half maximum of the resulting output neuron activity function as a simple measure of the intensity-specificity of the memory trace. We systematically vary one selected parameter at a time (red) and adjust all other parameters (grey) so as to keep this intensity-specificity at unity, mimicking the behavioural situation in Figure 1C. We then plot the resulting ranges of all parameters. Filled *vs.* open squares indicate the maximal *vs.* minimal value of the selected parameter and the corresponding values of the other parameters. This analysis highlights the relation between parameters, in particular between the potencies of homeostatic plasticity and inhibition: When *α* is raised, rendering homeostatic adjustment stronger, an increase in inh_max_ makes the inhibitory neuron more active. In turn, when inh_max_ is raised, this is compensated by an increase in *α*. In accordance with these findings, *α* and *a*_inh_ have an inverse relationship. Larger values of *α* (stronger homeostatic plasticity) are compatible with smaller values of *a*_inh_, reflecting a more sensitive inhibitory neuron. (C) Comparison of the results in (B) shows that all parameters can be varied over large ranges without perturbing the model output, as long as other parameters are adjusted appropriately.
